# The analgesic effect of propofol associated with the inhibition of hypoxia inducible factor and inflammasome in complex regional pain syndrome

**DOI:** 10.1186/s12929-019-0576-z

**Published:** 2019-10-18

**Authors:** Hung-Tsung Hsiao, Yuan-Yuarn Liu, Jeffrey Chi-Fei Wang, Ya-Chi Lin, Yen-Chin Liu

**Affiliations:** 1 Department of Anesthesiology, National Cheng Kung University Hospital, College of Medicine, National Cheng Kung University, Tainan City, 701 Taiwan; 2 Department of Emergency Medicine and Surgery, Kaohsiung Veterans General Hospital, Kaohsiung City, Taiwan

**Keywords:** Propofol, Chronic post-ischemic pain, Free radical, Hypoxic induced factor-1, Inflammasome

## Abstract

**Background:**

Complex regional pain syndrome (CRPS) is related to microcirculation impairment caused by tissue hypoxia and peripheral cytokine overproduction in the affected human limb and chronic post-ischemic pain (CPIP) is considered as an animal model for this intractable disease. Previous studies suggest that the pathogenesis of CPIP involves the hypoxia inducible factor-1α (HIF-1α) and an exaggerated regional inflammatory and free radical response. The inhibition of HIF-1α is known to relieve CPIP. So, propofol, as a free radical scavenger, is very likely to be beneficial in terms of relieving CPIP.

**Methods:**

We set up a CPIP model using the hindpaw of mice. We administered propofol (10 mg/kg) just after the reperfusion period (early stage) and also on the second day (late stage), as treatment. The analysis evaluated the expression of HIF-1α, free radicals, and inflammasome.

**Results:**

Propofol administration produced obvious analgesia in both mechanical and thermal evaluation in the early stage of CPIP (2 h after reperfusion). Only a mild analgesic effect was found in the late stage (48 h later after reperfusion). In the early stage, the expression of HIF-1α and the inflammasome marker (NALP1) along with caspase-1 were suppressed by propofol. The free radical level also decreased in the propofol group. But those molecular changes were not founded in the late stage of CPIP.

**Conclusion:**

Our data demonstrated that propofol produces mice analgesia in the early stage of CPIP and this effect is associated with inhibition of free radical, hypoxia inducible factor and inflammasome.

## Background

Chronic post-ischemic pain (CPIP)---caused by reperfusion injury---is due to vasoconstriction, tissue hypoxia, and generated cytokines in an affected limb. Previous studies suggested that CPIP includes exaggerated regional inflammatory and hypoxia responses to reperfusion injury [6]. Meanwhile, hypoxia-inducible factors (HIFs)---the transcription factors that respond to oxygen changes---have evidence to fortify the complex regional pain syndrome (CRPS), particularly during the acute phase. Previous studies also implied that the mice CPIP model, which is similar to human CRPS type I, includes exaggerated regional inflammation and HIF activation [6, 32]. Furthermore, in clinical, ischemia reperfusion (IR) injury results in tissue damage following a limb orthopedic operation when tourniquet applied. Because an IR injury is induced by hypoxic conditions, it is reasonable to consider HIF as the key to regulate this intractable pain. HIFs are transcription factors that respond quickly to microvascular environment during hypoxia [11]. It was also reported that the production of reactive oxygen species (ROS) responsible for HIF-1α expression under hypoxic conditions [3, 4] and antioxidants abolish this HIF response. Haddad et al. reported that ROS scavengers can stabilize HIF-1α in a concentration-dependent manner [10]. The ROS-induced inflammasome activation also triggers more ROS production and is crucial for caspase-1 processing and IL-1β secretion [25]. In other way, nucleotide-binding oligomerization domain-like receptors (NOD-like receptors, NLRs) are associated with cell stress. Although the NLR family are complex, the activation of the Nacht Leucine-rich-repeat Proteins (NALP) results in caspase activation [7]. Caspase-1 is the active enzymatic component of NALP1 inflammasome that cleaves pro-interleukin-1β to interleukin -1β (IL-1β) and then induces nociceptive sensitization. According to above evidence, the exogenous administration of antioxidant drugs during a reperfusion phase may theoretically attenuate inflammasome and cytokine production in IR injury.

The antioxidant capabilities of propofol, an anesthetic agent, were first reported in 1991 [23]. It is an ROS scavenger with anti-inflammation effects [27, 28, 30]. Propofol has also been reported to suppress proinflammatory cytokine [26] and to reduce LPS-induced ROS production via inhibition of inflammatory factors [13, 21]. We already proved that the HIF-1α inhibitor evokes analgesia and is associated with IL-1β reduction in a CPIP model [12]. Herein, we hypothesize that administration of propofol produce analgesic effect via ROS reduction, and then suppresses the expression of HIF-1α and inflammasome in CPIP mice.

## Methods

### Animals

Swiss male CD1 mice (7–8 weeks old, 25–30 g, from the Animal Center of National Cheng Kung University, Taiwan) arrived 7 days before the experiments. All animal experiments and procedures were carried out in accordance with the Animal Care Guidelines of National Cheng Kung University Medical College (IACUC approval No: 105259), Taiwan.

### Chronic post-ischemic pain model

The CPIP model was induced via a 3-h hindpaw IR injury, as described before [12]. Briefly, after anesthesia induction (isoflurane 1–2%), a Nitrile 70 Durometer O-ring with a 5/6 in. internal diameter was placed around the mouse’s left ankle joint for 3 h. The mice were anesthetized for the entire 3-h ischemia period under isoflurane (0.5–1.0%).

### Behavioral analysis and drug administration

The mice were habituated to the testing environment for at least 2 days before basal testing. Room temperature and humidity were controlled throughout the experiments. For mechanical sensitivity testing, the animals were placed on an elevated metal-mesh floor for over 30 min before examination. After the 3-h IR was completed and the O-ring removed, 10 mg/kg of propofol (B. Braun, Melsungen, Germany) was administered intravenously through tail vein of the mice for propofol group, 10% 200 μl of lipofundin for lipofundin group (as control solvent), and 200 μl of phosphate-buffered saline (PBS) for sham group, respectively. After injection, we tested the mice for mechanical allodynia using von Frey hair. The paw was pressed for 1 s with each hair using a series of them perpendicular to the plantar surface. The stiffness in the series was incremented along a logarithmic scale (0.02–2.56 g; Stoelting). The 50% withdrawal threshold was determined by Dixon’s up–down method as described in [5]. On day 2 after CPIP, Propofol 10 mg/kg was again injected intravenously to test the analgesic effect during late-stage of CPIP.

Heat sensitivity was also tested via radiant heat (55 °C) using a Hargreaves apparatus (7370; Ugo Basile). The basal latencies were adjusted by about 10–12 s, with a maximum cut-off value of 20 s to avoid tissue damage. The latencies were averaged over three trials, separated by 3–5 min intervals, as described before [20].

### Western blot analysis

The animals were killed rapidly after deep anesthesia with isoflurane (5%) when sampling. The left dorsal horn of the spinal cord over lumbar enlargement and the paw skin tissues from the lesion side (left) of the hind limbs were quickly removed and homogenized for 2 h or 2 days after CPIP. The samples were stored at 4 °C, and homogenized mechanically in a tissue buffer containing 50 mmol/L Tris-HCl, 150 mmol/L NaCl, 1 mmol/L EDTA, 1% Igepal (SigmaAldrich), 1% sodium deoxycholate, and 0.1% SDS (pH 7.4). The homogenates were centrifuged at 9000 rpm for 20 min, then the supernatants were collected and subjected to western blotting for the detection of endogenous HIF-1α. The SDS sample buffer contained a mixture of proteinase and phosphatase inhibitors (Sigma). The protein samples (30 μg in 25 μL) were separated in a sodium dodecyl sulfide–polyacrylamide gel electrophoresis (SDS–PAGE) gel, and transferred to nitrocellulose blots. SDS-PAGE was performed under the reduced conditions of 4-12% mini-gels in precast with the NuPAGE Bis-Tris buffer system (Invitrogen, Carlsbad, CA). The blots were blocked with 5% milk, and incubated overnight at 4 °C with primary antibodies against HIF-1α and IL-1β. These primary antibodies were used as an anti-human HIF-1α mouse monoclonal antibody (1:500, Sigma-Aldrich) and IL-1β (1:1000, cat# ab9722, Abcam, Cambridge, MA, USA), (active IL-1β, 17 kD). NALP1 was examined using rabbit anti-NALP1 (1:500, cat# 4990, Cell Signaling), while caspase-1 was examined using rabbit anti-caspase-1 (diluted 1:1500, cat# ab108362, Abcam). In similar manner, we used Rabbit anti-NALP3 (diluted 1:500, cat# MAB7578, R&D), rabbit anti-NF-κB (diluted 1:2000, cat# 622602, BioLegend), rabbit anti-IκB-α (diluted 1:2000, cat# GTX110521, GeneTex), and rabbit anti-TNF-α (diluted 1:1500, cat# GTX110520, GeneTex). Endogenous β-actin was used as loading control with the anti-human β-actin mouse monoclonal antibody (cat# MAB1501, EMD Millipore, Billerica, CA, USA). These blots were further incubated with HRP-conjugated secondary antibody, developed in ECL solution, and were exposed on hyperfilm (Amersham Biosciences, Little Chalfont, Bucks, UK) for 1–10 min. Subsequently, the specific bands were evaluated according to apparent molecular size---the intensities of which were captured and analyzed using Image J software (NIH, Bethesda, MA, USA).

### Measurement of ROS and Myeloperoxidase (MPO)

The levels of ROS and MPO were measured for comparison with formerly documented mechanisms [31]. Briefly, a lucigenin-based chemiluminescence assay was used to detect ROS production. Plantar tissues from the hindpaw of the mice were harvested and weighed serially to investigate the ROS trend and the impact of propofol. Homogenized skin tissues were isolated with trypsin EDTA, centrifuged, and their supernatant collected. Subsequently, 1 ml Tyrode’s solution (Calcium free) was added. The pellet was mixed well and obtained immediately for O_2_-measurement. The fluorescence values were read immediately: at a wavelengths of 485 nm for excitation and at 530 nm for emission. Chemiluminescence was measured for 2.5 min at room temperature with a luminometer (Siriusluminometer, Berhold, Germany). O_2_-production was calculated and converted to percentage for controlling. MPO was measured to identify the neutrophil activity in the skin. Tissue, after being weighed, was lysed in a triton X-100 0.05%, and kept frozen at − 70 °C until use. The substrate cocktail for the assay was prepared with 5 ml of 0.1 M citrate buffer, 32 μl of Triton X-100 20%, 50 μl of 82.4 mM O-Dianisidine in dimethyl sulfoxide, and 20 μl of 26.4 mM H_2_O_2_. 0.7 ml of 0.1 M citrate buffer (pH 5.5) was added to 0.5 ml of cell lysate. Then, 4.8 ml of the above assay mixture was added at room temperature for 1 h. OD_450_ was used to measure the MPO intensity.

### Statistical analysis

The results were expressed as mean along with the standard error of the mean (SEM). A two-way analysis of variance (ANOVA), Student’s t-test (two groups), and multiple comparisons were used for statistical evaluation of the differences among means. A Newman–Keuls test was used for post hoc analysis. The value of *p* < 0.05 was considered to indicate a statistically significant difference.

## Results

### Measurement of mechanical allodynia and thermal hyperalgesia

**Fig. 1:**
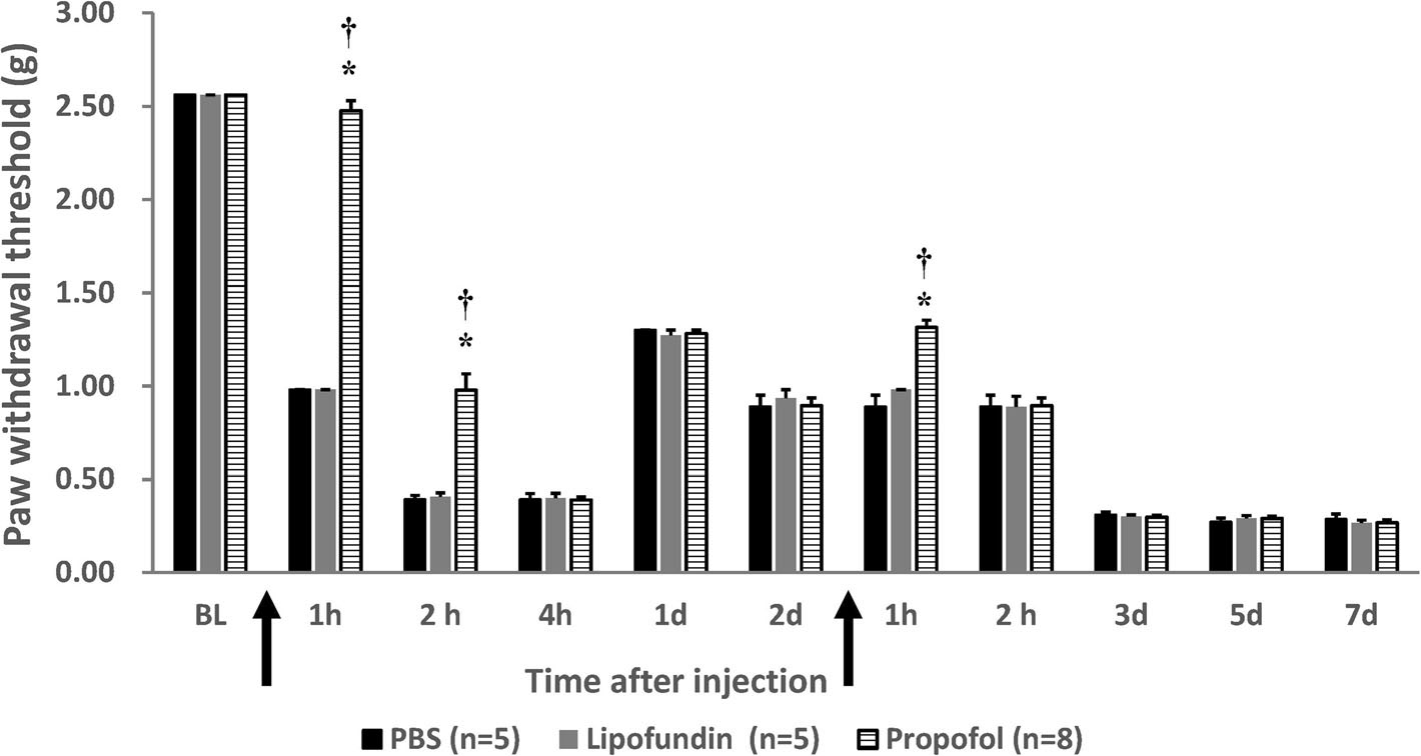
Measurement of mechanical allodynia of the mice. Behavioral response (anti-allodynia) of the mice in our chronic post-ischemic pain (CPIP) model treated with propofol (10 mg/kg, PBS & lipofundin as control), single dose. ↑ represents the injection time point. The X-axis represents the timeline (BL: basal level; 1 h, 2 h, 4 h: 1, 2, and 4 h after CPIP O-ring removal and drug injection; 1 d, 2 d, 3 d, 5 d, 7 d: 1, 2, 3, 5, and 7 days after CPIP was induced.) Values are presented as mean ± SEM, *n* = 5–8 per group. In the Student’s t-test, * represents *P* < 0.05 vs. the CPIP PBS group, and † represents *P* < 0.05 vs. the lipofundin group

**Fig. 2:**
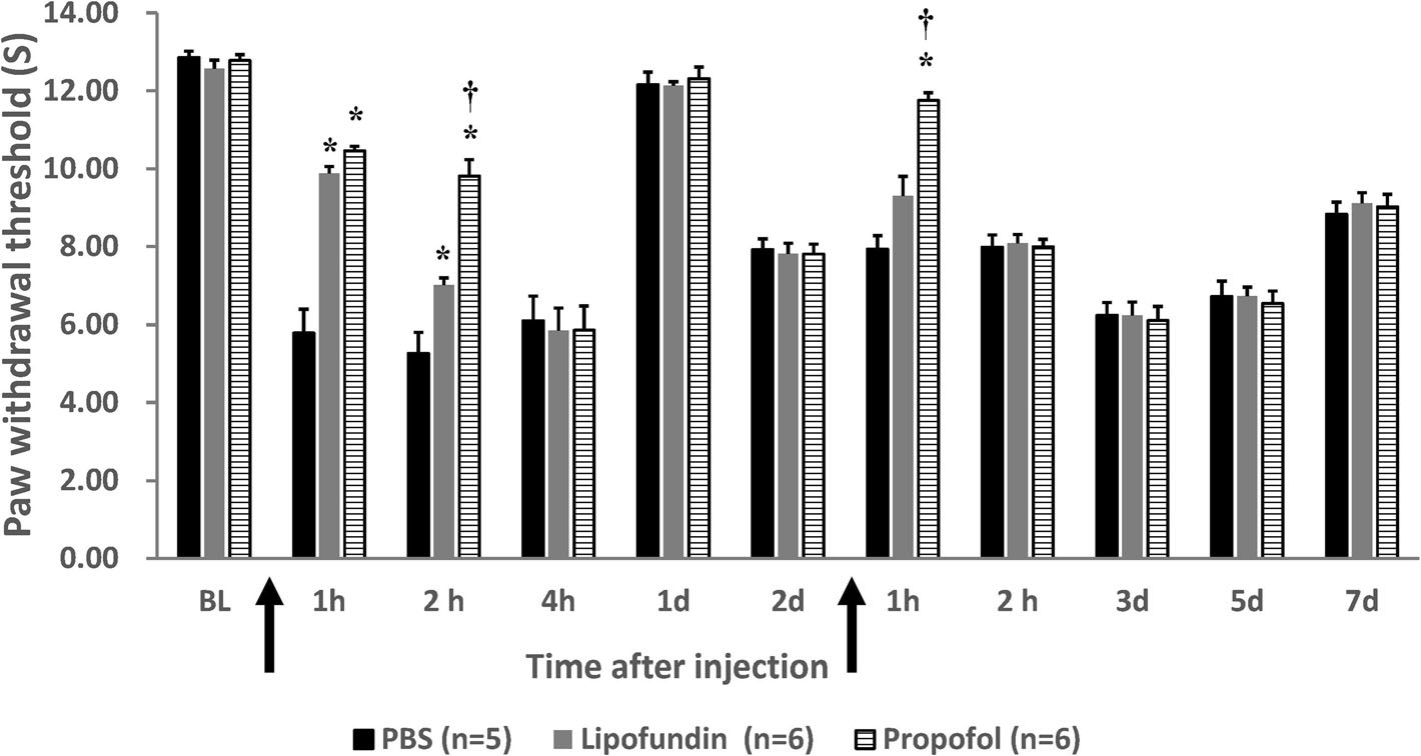
Measurement of thermal hyperalgesia of the mice. Behavioral response (thermal hyperalgesia) for mice in the CPIP model treated with propofol (10 mg/kg, PBS & lipofundin as control), single dose. ↑ represents the injection time point. The X-axis represents the time course (BL: basal level; 1 h, 2 h, 4 h: 1, 2, and 4 h after CPIP O-ring removal and drug injection; 1d, 2d, 3d, 5d, 7d: 1, 2, 3, 5, and 7 days after CPIP was induced.) Values are presented as mean ± SEM, *n* = 5–6 per group. Student’s t-test,* represents *P* < 0.05 vs. the CPIP PBS group and † represents *P* < 0.05 vs. the lipofundin group

We administered propofol immediately after removing the O-ring, and repeated 48 h later. Figure 1 displays the results about mechanical allodynia along the time line. The withdrawal thresholds clearly show the effect of propofol compared with those in the PBS and lipofundin groups. The significant differences appear during the initial first 2 h of CPIP. On the initial second hour, we measured: 0.98 ± 0.09 (propofol), 0.41 ± 0.02 (lipofundin), and 0.39 ± 0.02 (PBS). On the first hour on day 2, we measured: 1.32 ± 0.11 (propofol), 0.98 ± 0.01 (lipofundin), and 0.89 ± 0.13 (PBS). In summary, there were statistically significant differences between the measurements of the propofol group and those of the other two after the first and the second hours on day 1. Significant difference was also noted during the first hour on day 2. Figure 2 shows the thermal hyperalgesia withdrawal threshold for the CPIP mice. Propofol produced an obvious analgesic effect compared to that in the sham group, and a better result than that in the lipofundin group. On the initial second hour, we measured: 9.82 ± 0.42 (propofol), 7.01 ± 0.18 (lipofundin), and 5.26 ± 0.54 (PBS).

### ROS from harvested plantar tissues

**Fig. 3:**
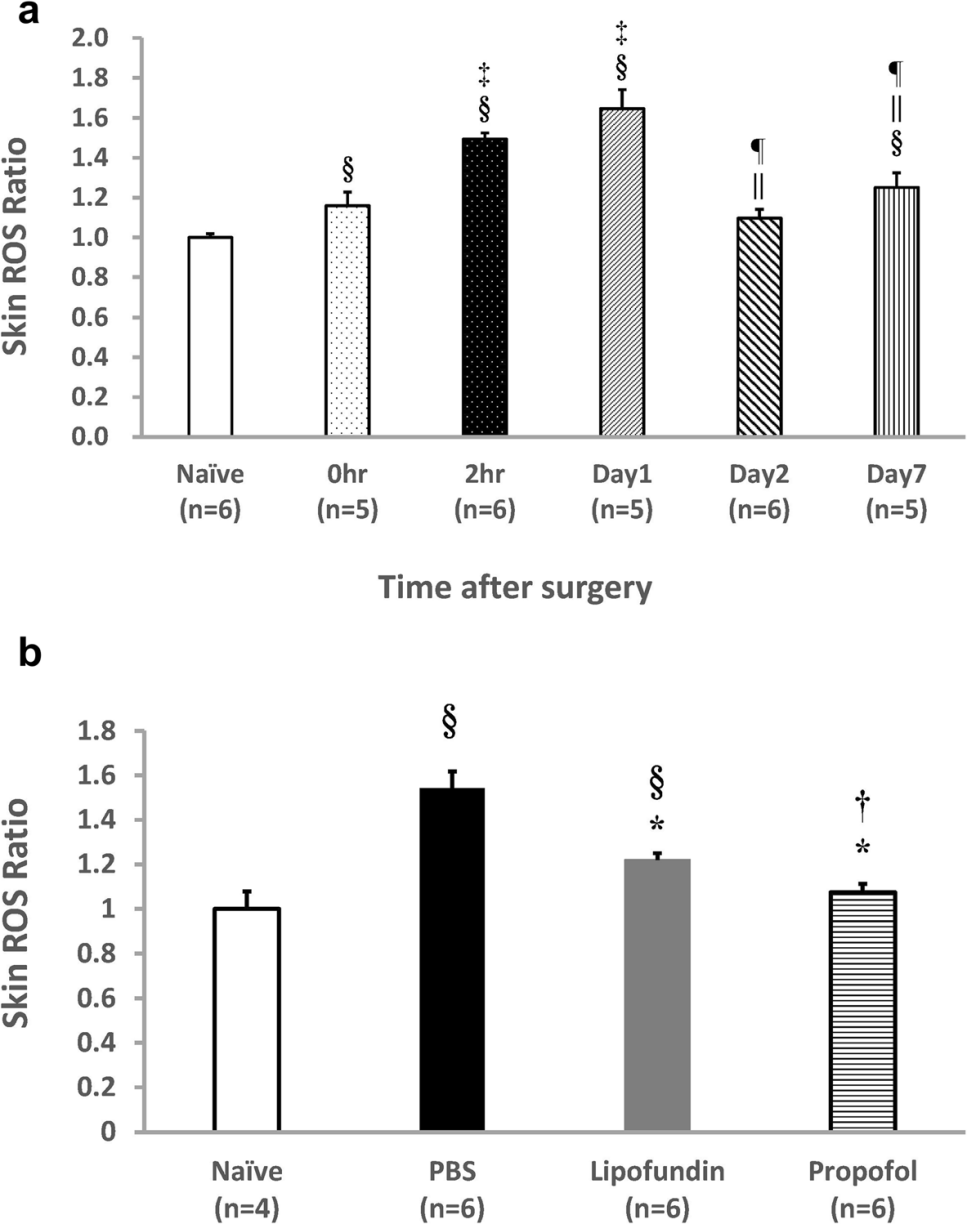
ROS of hindpaw plantar tissue. The serial changes of ROS in plantar tissue (**a**) and the impact of propofol (**b**) are presented. ROS production was measured via chemiluminescence. Values are displayed as mean ± SEM, *n* = 4–6 per group. **a** The level of ROS in CPIP were increased from 1.00 ± 0.02 (Naïve) to 1.49 ± 0.03 (2 h after CPIP) to 1.65 ± 0.09 (1 day after CPIP) then decreased to 1.10 ± 0.05 (2 days after CPIP) to 1.25 ± 0.07 (7 day after CPIP). In the Student’s t-test, § represents *P* < 0.05 vs. the Naïve group, ‡ represents *P* < 0.05 vs. the 0 h group, || represents *P* < 0.05 vs. the 2 h group, ¶ represents *P* < 0.05 vs. the Day 1 group. **b** The impact of propofol on ROS (2 h after CPIP) was 1.00 ± 0.08 (Naïve) to 1.54 ± 0.08 (PBS group) and decreased to 1.07 ± 0.04 (propofol group). * represents *P* < 0.05 vs. PBS group, † *P* < 0.05 vs. lipofundin group, § *P* < 0.05 vs. Naive group

The serial level of ROS from the hindpaw plantar tissues and the ROS level of the four groups 2 h after CPIP are presented in Fig. 3(a)(b). The serial ratio of ROS increased significantly from 1.00 ± 0.02 (Naive) to 1.49 ± 0.03 (2 h after CPIP). One day after CPIP initiation, it increased to 1.65 ± 0.09. Two days after CPIP, it decreased to 1.10 ± 0.05 but increased to 1.25 ± 0.07 on the seventh days after CPIP (Fig. 3a). The comparative ratios of ROS (2 h after CPIP) to the naive group were the following: 1.54 ± 0.07 for the PBS group, 1.22 ± 0.03 for the lipofundin group, and 1.07 ± 0.04 for the propofol group (Fig. 3b). Propofol had a significant inhibition on ROS expression.

### Protein expression of HIF-1α, inflammasome, and cytokines in plantar tissues of hindpaws and the spinal cord

**Fig. 4:**
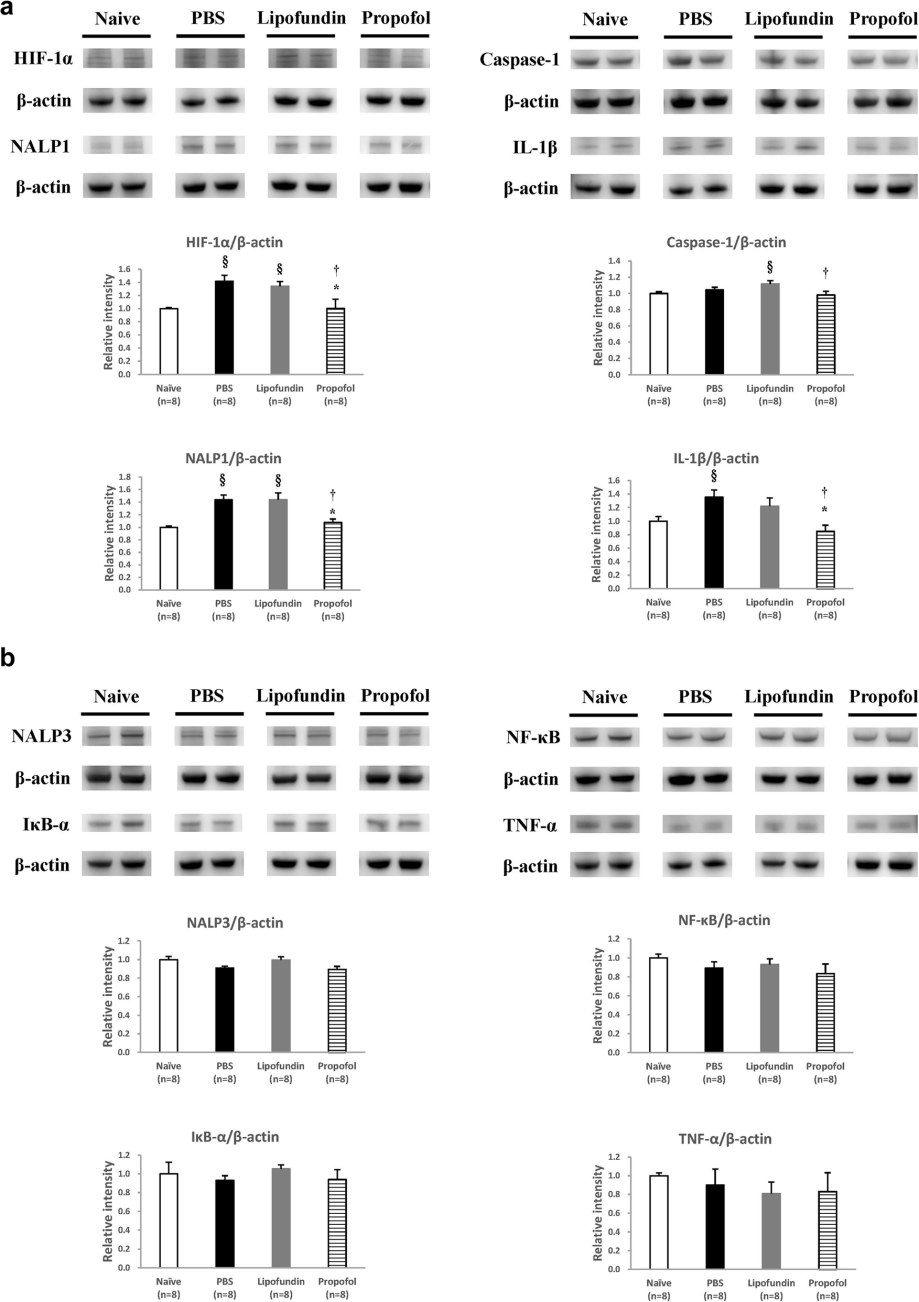
Western Blot of plantar tissues with drugs given 2 h after CPIP. **a** Representative data for the western blots of HIF-1α, NALP1, Caspase-1, and IL-1β in paw skin. Values are presented as mean ± SEM, *n* = 8 per group. Student’s t-test, § represents *P* < 0.05 vs. the Naïve group, * represents *P* < 0.05 vs. the PBS group, † represents *P* < 0.05 vs. the lipofundin group. **b** Representative data for the western blots of NALP3, NF-κB, IκB, and TNF-α in paw skin. Values are presented as mean ± SEM, *n* = 8 per group. Student’s t-test

**Fig. 5:**
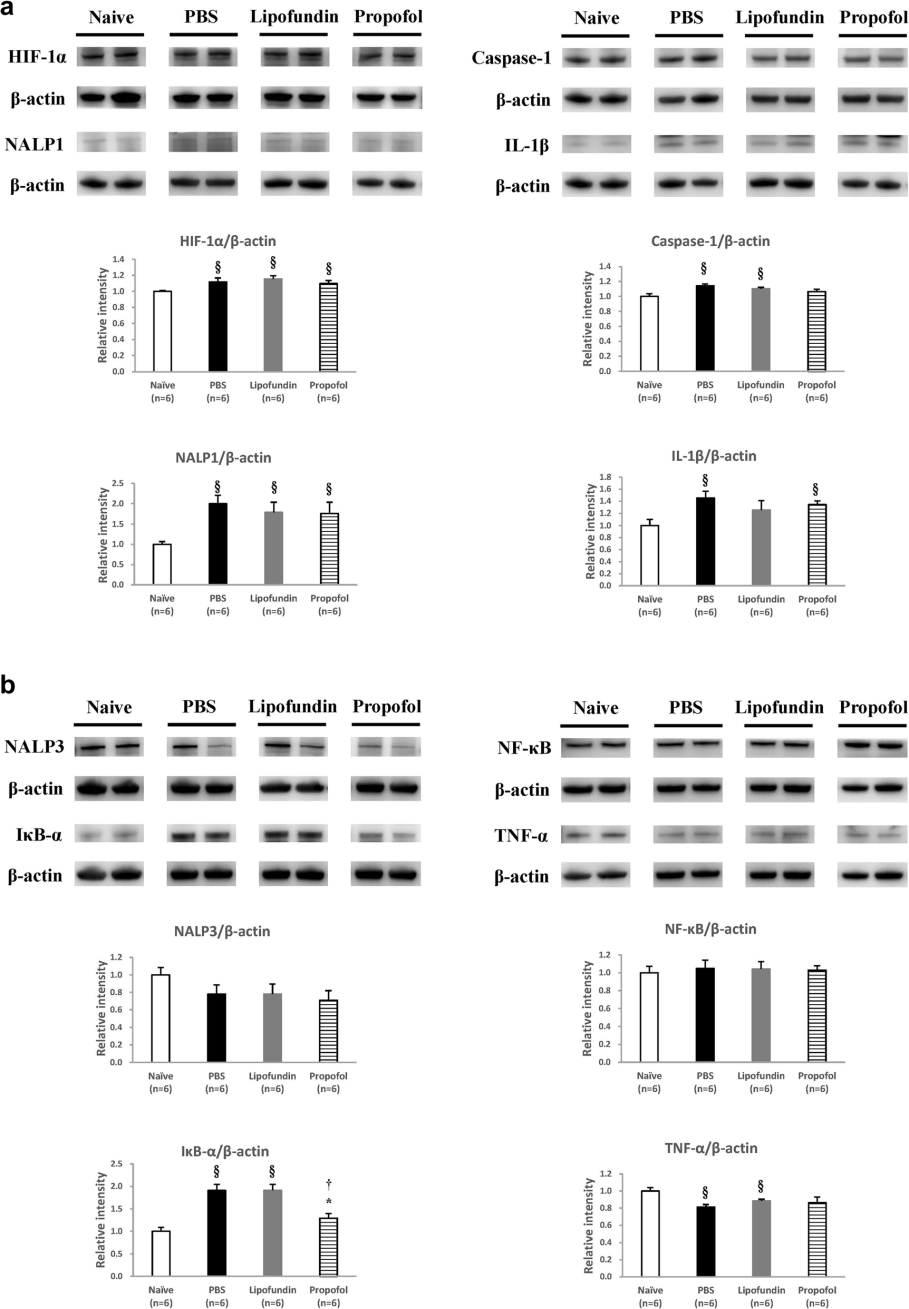
Western Blot of plantar tissues 2 days after CPIP. **a** Representative data for the western blots of HIF-1α, NALP1, Caspase-1, and IL-1β in paw skin. Values are presented as mean ± SEM, *n* = 6 per group. Student’s t-test, § represents *P* < 0.05 vs. the Naïve group. **b** Representative data for the western blots of NALP3, NF-κB, IκB, and TNF-α in paw skin. Values are presented as mean ± SEM, *n* = 6 per group. Student’s t-test, § represents *P* < 0.05 vs. the Naïve group, * represents *P* < 0.05 vs. the PBS group, † represents *P* < 0.05 vs. the lipofundin group

Expression of HIF-1α, inflammasome, and cytokines in the plantar tissues of the hindpaws are presented in Fig. 4 (2 h after CPIP) and in Fig. 5 (2 days after CPIP). According to the behavior data, 2 h after CPIP has behavior difference both on mechanical and thermal measurement. We also considered the effect of control solvent (lipofundin) and sampling on the 2 h after CPIP. HIF-1α, NALP1, caspase-1, and IL-1β are presented in Figs. 4a and 5a. NALP3, NF-κB, IκB, and TNF-α are presented in Figs. 4b and 5b.

#### Two hours after CPIP (Fig. 4)

Densitometric analysis of the western blot bands demonstrated that the expression of HIF-1α and IL-1β significantly decreased by the use of propofol (10 mg/kg) compared to the two control groups, see Fig. 4a. The expression of NALP1 and Caspase-1 was also obviously inhibited in the propofol group (NALP1/β-actin ratio = 1.08, caspase-1/β-actin ratio = 0.98) compared to the PBS group (NALP1/β-actin ratio = 1.43, caspase-1/β-actin ratio = 1.04), as shown in Fig. 4a. The production of IL-1β in the PBS group was significantly higher (1.4-fold) than that in the naive group. The HIF-1α, NALP1, and IL-1β productions in the PBS group were also statistically higher than those in the naive group, which indicates that the painful component of CPIP is related to inflammasome. HIF-1α may be the triggering factor. However, other inflammasomes, including NALP3, NF-κB, IκB, and TNF-α, were not affected by the CPIP model, and there was no change after propofol administration, either (Fig. 4b). This means that various inflammasomes play different roles in this CPIP model.

#### Two days after CPIP (Fig. 5)

In a clinical setting, a patient presenting IR induced pain may receive treatment several days later. Therefore, we also administered propofol 2 days after CPIP to investigate propofol effect. We also harvested plantar tissues of the hindpaws one hour after propofol injection (10 mg/kg). However, the protein expressions of HIF-1α, IL-1β, NALP1, and Caspase-1 were not affected after any propofol administration (Fig. 5a). Again, we found no statistical difference in the levels of NALP3, NF-κB, and TNF-α between the 3 groups (Fig. 5b). However, the IκB level was significantly higher than that in the naive group and propofol suppressed its expression on day 2.

#### Inflammasome in spinal cord after CPIP (Fig. 6)

**Fig. 6:**
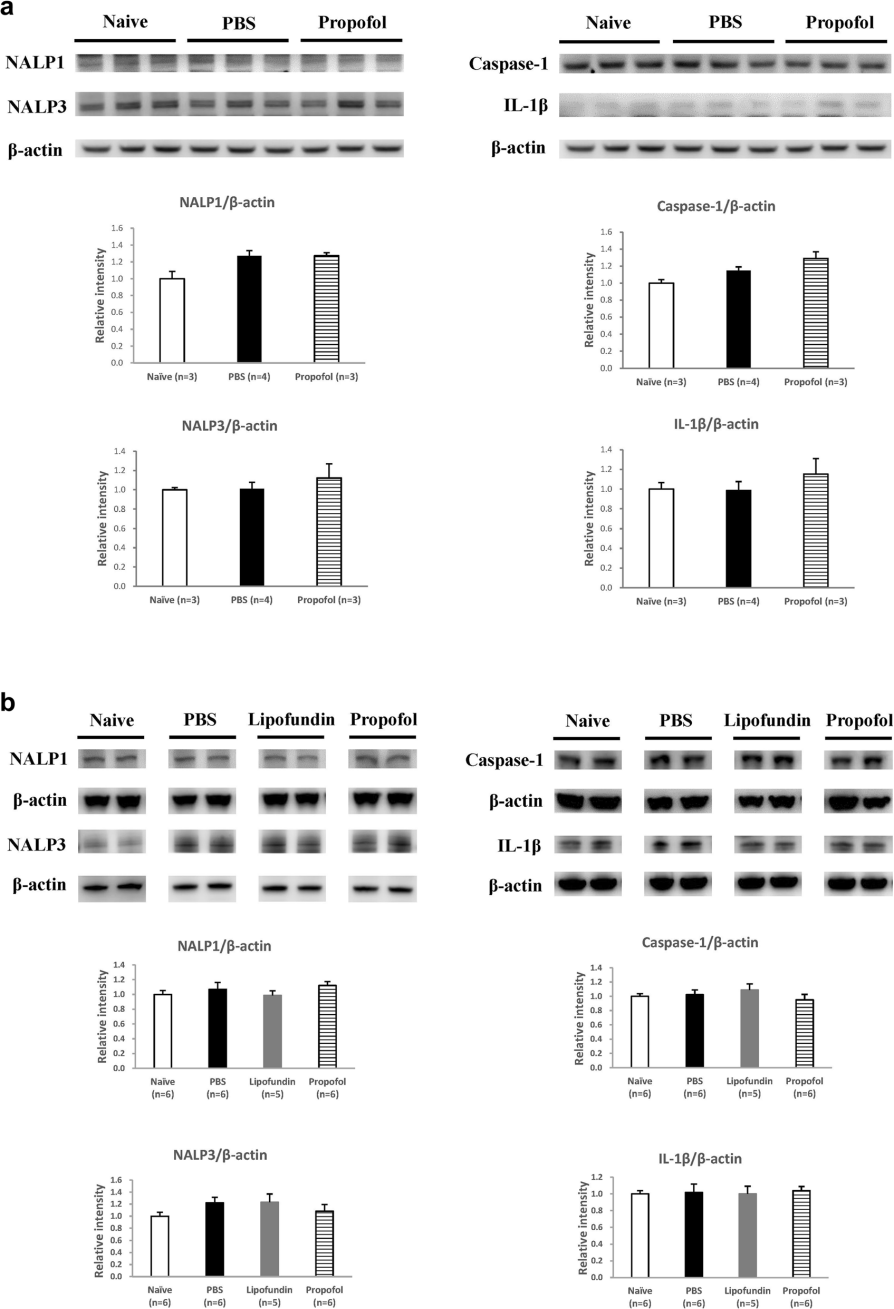
Western Blot of spinal cord tissues 2 h after CPIP and day 2 after CPIP. Representative data for the western blots of NALP1, Caspase-1, NALP3, and IL-1β in the ipsilateral dorsal horn of the spinal cord 2 h after CPIP (**a**) and day 2 after CPIP (**b**). Values are presented as mean ± SEM. No statistical difference (*P* < 0.05) was found

We also harvested spinal cord tissues (only ipsilateral dorsal horn) at the same time point as plantar skin tissue. The western blots of NALP1, Caspase-1, NALP3, and IL-1β in the spinal cord 2 h after CPIP (Fig. 6a) and 2 days after CPIP (Fig. 6b) showed no statistical difference between the groups.

### MPO from harvested plantar tissues

**Fig. 7:**
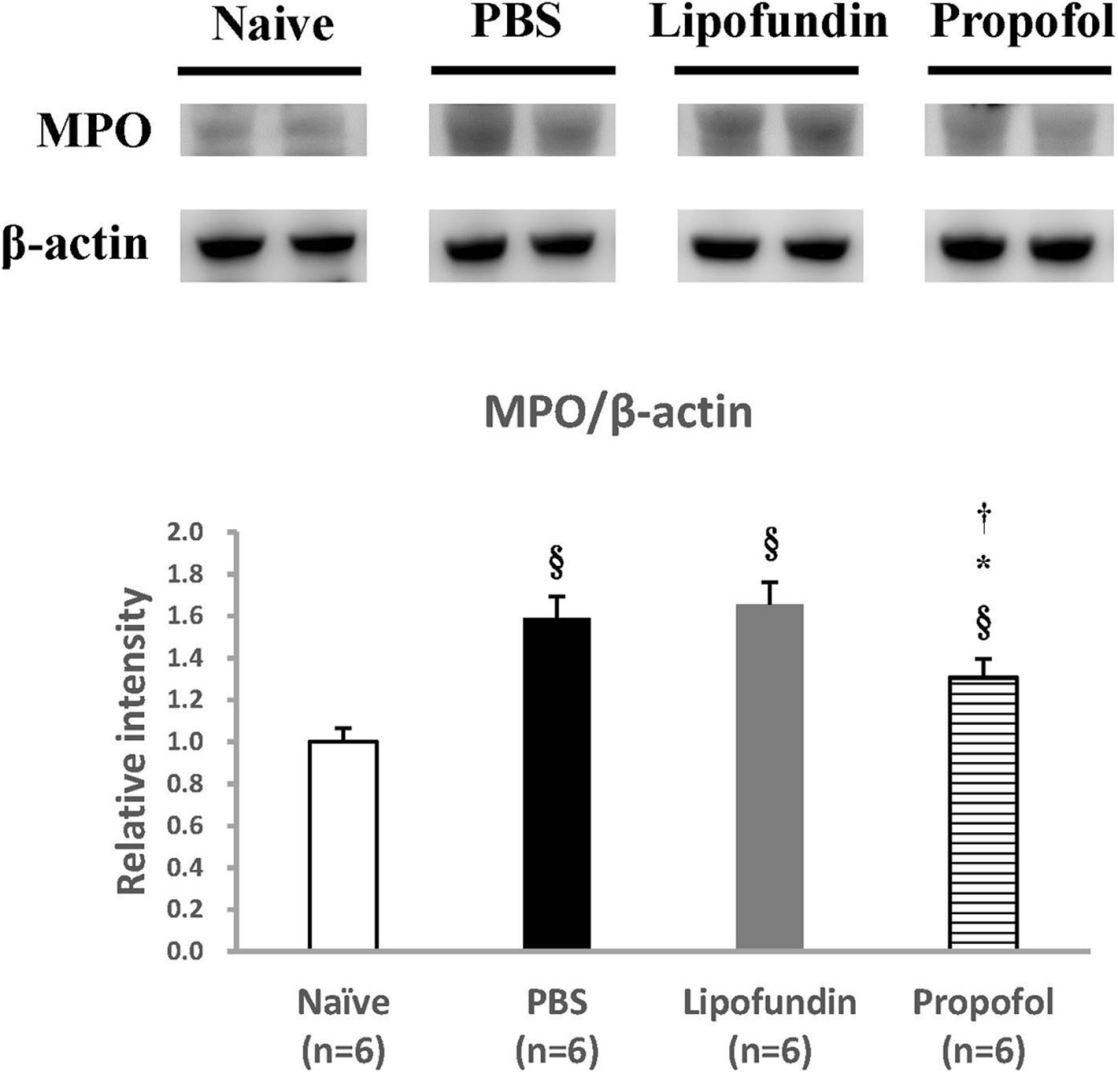
Myeloperoxidase (MPO) activity of plantar tissues 2 h after CPIP. The MPO level was significantly increased and decreased by propofol. Propofol significantly suppress macrophage activity Values are presented as mean ± SEM. Student’s t-test, § represents *P* < 0.05 vs. Naïve group, * represents *P* < 0.05 vs. PBS group, † represents *P* < 0.05 vs. lipofundin group

The MPO level comparison between the four groups 2 h after CPIP is presented in Fig. 7. The ratio of MPO significantly increased from 1.00 ± 0.05 (Naive) to 1.59 ± 0.08 (PBS group). It also increased to 1.66 ± 0.09 (lipofundin group), but decreased to 1.31 ± 0.07 (propofol group). Propofol had a significant suppression effect on macrophage activity.

## Discussion

**Fig. 8:**
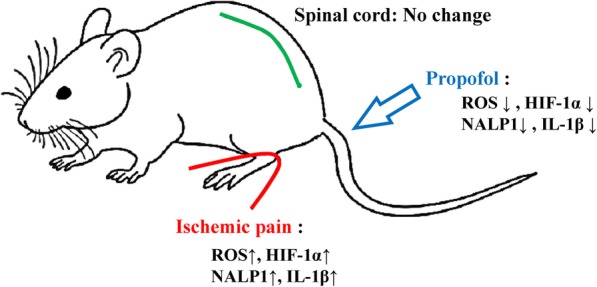
Graphic summary. In CPIP model, ROS plus HIF-1α, NALP1 and IL-1β increase and propofol administration can reduce ROS level and the expression of HIF-1α, NALP1 and IL-1β and then produced analgesia. Those effects were happened in plantar but not spinal cord

Our results demonstrated that propofol, an ROS scavenger, can reduce the symptoms of CPIP with its inhibition of HIF-1α, inflammasome, and cytokine. Under hypoxic conditions, HIF-1α production is an ROS enhancing activity that induces transcription and translation of HIF-1α [8, 15]. ROS also induces activating inflammasome of NALP1 and NALP3 as well as caspase-1 and IL-1 in macrophages. Those reactions are significantly inhibited by treatment with ROS inhibitors [9]. Propofol then can suppress those responses and produced better analgesia in CPIP mice (Fig. 8). Non-anesthetic effects of propofol have been reported on anti-inflammation and anti-oxidative stress [21, 22]. Propofol is also an anesthetic, thus it might have had residual amnesic effect on behavioral measurement after treatment. But if the amnesic effect of propofol had played a major role in behavior, behavioral data should be similar on both days of treatment (2 h and day 2 after CPIP initiation). We also demonstrated that the inflammasome response diminished, and also showed that propofol can suppress ROS production, decrease HIF-1α, and suppress NALP1 inflammasome along with caspase-1 (Figs. 3 and 4). It is reasonable to think that during CPIP, ROS and HIF accumulated inducing the inflammasome and cytokine reactions, which were all suppressed by propofol. The macrophage (MPO activity) maybe the target when propofol administration during CPIP.

Previous reports demonstrated that continuous administering of IL-1 receptor blocker reduces both nociceptive sensitization [18, 19] and peripheral inflammation [19]. It has also been found that NALP1 inflammasome containing caspase-1 can be activated in keratinocytes [18]. The activation of NALP1 and caspase-1 proteins is related to neuropathic pain [17]. We didn’t overlook that keratinocytes might play a crucial role in CPIP models. Further studies are needed to resolve this issue.

Propofol administration on the second day didn’t produce better analgesia than that obtained initially. Our explanation is that CPIP provokes an HIF-1α reaction that can last seven days [12]. CPIP model is a complex progressive process with persistent pain for 7 to 10 days and induces many molecules once activated. This model have two phase of pain presentation: 1.acute stage: happen just after reperfusion injury and last for several hours and then gradually better. 2. Second stage: happen around the second day and persistent worse to Day 7–10 and gradually recovery on Day 10–14. Pain mechanism may be different in those two stages [12]. If the inflammatory cascade other than ROS is induced on the second day, propofol administration cannot produce better analgesia than what was obtained initially. Fortunately, propofol, as a short term anesthetic, can be infused continuously during an operation. We predict that the continuous infusion of propofol may produce less post-operative IR-induced pain. Certainly, more evidence is needed to formulate and prove this concept.

According to the results 2 h or 2 days after CPIP (Fig. 6), we concluded that, under CPIP condition, propofol (10 mg/kg iv) had no effect on inflammasome within the spinal cord. Although fewer animals were used on results of 2 h, we think that acute peripheral pain seldom induced central spinal change and we had proved that intrathecal injection of HIF inhibitor had no analgesia effect in acute stage of CPIP [12]. So we think, in the acute stage, central spine cord play little effect on the CPIP pain behavior. But it is also possible that inflammasome played a different role in spinal cord for this CPIP model or the concentration of propofol within spinal cord is not high enough to produce changes.

ROS is also critical for NF-κB activation [1, 14, 24]. Many reports demonstrated that NF-κB can be activated by ROS, and that propofol inhibits the NF-κB signal [2, 29]. In our research, NF-κB was not activated in the CPIP model at all (Fig. 4b). The role of NF-κB in a CPIP model may be different from that of other inflammasomes like NALP1 and caspase-1. Similar situations were noted about NALP3, IκB, and TNF-α. NALP3 and NALP1 proteins can assemble to inflammasomes that activate caspase-1, resulting in the processing of cytokines IL-1ß [16]. Theoretically, these inflammasomes and cytokines should be activated in this CPIP model. However, our results contradicted to this theory.

## Conclusion

This is the first experiment to demonstrate the association between inhibition of propofol on HIF-1α, NALP1, caspase-1 plus IL-1β expression, and propofol analgesic behavior in the initial CPIP stage. We also demonstrated that other inflammasomes, NALP3, NF-κB, and IκB plus cytokine TNF-α, are not affected by propofol administration and that early administration of propofol provides better analgesia in this CPIP pain model. We suggest that propofol should be used for clinical IR conditions if ROS and inflammasome were massively produced.

## Data Availability

The datasets used in the current study are available from the corresponding author upon request.
